# Glucose Availability and AMP-Activated Protein Kinase Link Energy Metabolism and Innate Immunity in the Bovine Endometrium

**DOI:** 10.1371/journal.pone.0151416

**Published:** 2016-03-14

**Authors:** Matthew L. Turner, James G. Cronin, Pablo G. Noleto, I. Martin Sheldon

**Affiliations:** 1 Institute of Life Science, Swansea University Medical School, Singleton Park, Swansea, United Kingdom; 2 Faculty of Veterinary Medicine, Federal University of Uberlândia, Uberlândia, Brazil; Hull York Medical School, UNITED KINGDOM

## Abstract

Defences against the bacteria that usually infect the endometrium of postpartum cattle are impaired when there is metabolic energy stress, leading to endometritis and infertility. The endometrial response to bacteria depends on innate immunity, with recognition of pathogen-associated molecular patterns stimulating inflammation, characterised by secretion of interleukin (IL)-1β, IL-6 and IL-8. How metabolic stress impacts tissue responses to pathogens is unclear, but integration of energy metabolism and innate immunity means that stressing one system might affect the other. Here we tested the hypothesis that homeostatic pathways integrate energy metabolism and innate immunity in bovine endometrial tissue. Glucose deprivation reduced the secretion of IL-1β, IL-6 and IL-8 from *ex vivo* organ cultures of bovine endometrium challenged with the pathogen-associated molecular patterns lipopolysaccharide and bacterial lipopeptide. Endometrial inflammatory responses to lipopolysaccharide were also reduced by small molecules that activate or inhibit the intracellular sensor of energy, AMP-activated protein kinase (AMPK). However, inhibition of mammalian target of rapamycin, which is a more global metabolic sensor than AMPK, had little effect on inflammation. Similarly, endometrial inflammatory responses to lipopolysaccharide were not affected by insulin-like growth factor-1, which is an endocrine regulator of metabolism. Interestingly, the inflammatory responses to lipopolysaccharide increased endometrial glucose consumption and induced the Warburg effect, which could exacerbate deficits in glucose availability in the tissue. In conclusion, metabolic energy stress perturbed inflammatory responses to pathogen-associated molecular patterns in bovine endometrial tissue, and the most fundamental regulators of cellular energy, glucose availability and AMPK, had the greatest impact on innate immunity.

## Introduction

Metabolic stress compromises inflammatory defence responses against bacterial infections of the uterus in cattle after parturition, but it is unclear how metabolism and immunity are integrated in the endometrium. There is emerging evidence that energy metabolism and innate immunity are integrated in tissues [1, 2]; so that if one system is stressed, the other is likely to be affected. Understanding how metabolism and immunity are integrated in the endometrium is important because failure to efficiently counter postpartum bacterial infections leads to uterine disease in up to 40% of dairy cattle; and, the cost of treatment, reduced milk production, and replacing infertile animals is €1.4 billion/year in the European Union [3].

The energetic demand of lactation is three times the daily resting metabolic rate, for a modern high-milk yield dairy cow [4]. Running 100 km every day is an equivalent energetic demand for humans. The dairy cow cannot consume enough food to meet the metabolic demand of lactation, leading to a “negative energy balance”. Consequently, there is reduced availability of glucose and of the endocrine regulator insulin-like growth factor-1 (IGF-1) in peripheral plasma [5, 6]. In addition, all dairy cattle have an invasion of the uterus with numerous Gram-negative and Gram-positive bacteria after parturition [7, 8]. Negative energy balance is associated with persistence of these infections, leading to uterine disease [6, 9, 10]. However, little is known about mechanisms that link energetic stress and immunity in the endometrium.

Cellular energy homeostasis depends on multiple systems, including glucose uptake via plasma membrane glucose transporters, production of ATP from glucose via glycolysis and the Krebs cycle, and the control of cellular metabolism by AMP-activated protein kinase (AMPK) and mammalian target of rapamycin (mTOR) [11, 12]. Glucose transporters are widely distributed in the uterus of most species, and some are regulated by ovarian steroids and insulin [13]. Glucose transporters are also expressed in the bovine endometrium, and particularly by epithelial cells, but they are not regulated by ovarian steroids [14]. The principal intracellular regulator of energy homeostasis is AMPK, which senses increased AMP to ATP ratios associated with metabolic stresses, such as reduced glucose availability and hypoxia, or increased glucose and ATP consumption by biosynthetic pathways [11]. Activation of AMPK, by phosphorylation, restores energy balance by inhibiting anabolic pathways that consume ATP, whilst stimulating glycolysis, mitochondrial biogenesis, and expression of glucose transporters [11, 15]. On the other hand, mTOR integrates multiple signals that reflect the availability of metabolic energy, amino acids, and IGF-1 [12, 16]. When nutrients are abundant, activated mTOR complexes stimulate anabolic pathways and cell growth. Conversely, if energy is limiting, phosphorylated AMPK inhibits mTOR activity to conserve energy and reduce protein synthesis. Metabolic stress can also induce aerobic glycolysis, known as the Warburg effect, where cells rapidly generate ATP and carbon substrates from glucose flux through glycolysis rather than the Krebs cycle [17, 18]. Interestingly, innate immune responses to bacteria induce the Warburg effect in murine macrophages [19].

Innate immunity is predicated on cellular pattern recognition receptors, such as Toll-like Receptors (TLRs), binding to pathogen-associated molecular patterns [20, 21]. For example, TLR4 binds the lipopolysaccharide (LPS) component of Gram-negative bacteria, whilst TLR2 binds bacterial lipopeptides. The binding of pathogen-associated molecular patterns to TLRs leads to the release of cytokines and chemokines, such as interleukin (IL)-1β, IL-6 and IL-8, which attract and regulate hematopoietic immune cells, to clear the invading microbes [20–22]. However, this innate immune response needs to be rapid and vigorous enough to efficiently eliminate microbes from tissues, otherwise chronic inflammation may develop [22]. The inflammatory responses to postpartum bacterial infections of the bovine endometrium are typical of innate immunity, with increased abundance of IL-1β, IL-6 and IL-8 [23–25]. Furthermore, endometrial tissue and cells express TLR4 and TLR2, and mount inflammatory responses to LPS and lipopeptides *in vitro* [26–29].

It has been suggested that negative energy balance impairs the inflammatory response and clearance of bacteria from the endometrium, leading to chronic endometritis [6, 10]. This view is contentious because induced negative energy balance does not impair innate immune responses to bacteria in the mammary gland of cattle [30]. To explore this conundrum about interactions between energy metabolism and innate immunity, we used a reductionist approach with *ex vivo* organ cultures of bovine endometrium, which secrete cytokines and chemokines in response to pathogen-associated molecular patterns, similar to the tissues *in vivo* [28, 29]. As we aim to study responses by tissues, organ cultures have advantages over isolated cells because organ cultures maintain tissue architecture, whilst avoiding potential confounders of *in vivo* studies, including humoral factors, adaptive immune responses, and endocrine status. The present study tested the hypothesis that homeostatic pathways integrate energy metabolism and innate immunity in the bovine endometrium.

## Results

### Inflammatory responses are blunted by glucose deprivation

**Fig 1:**
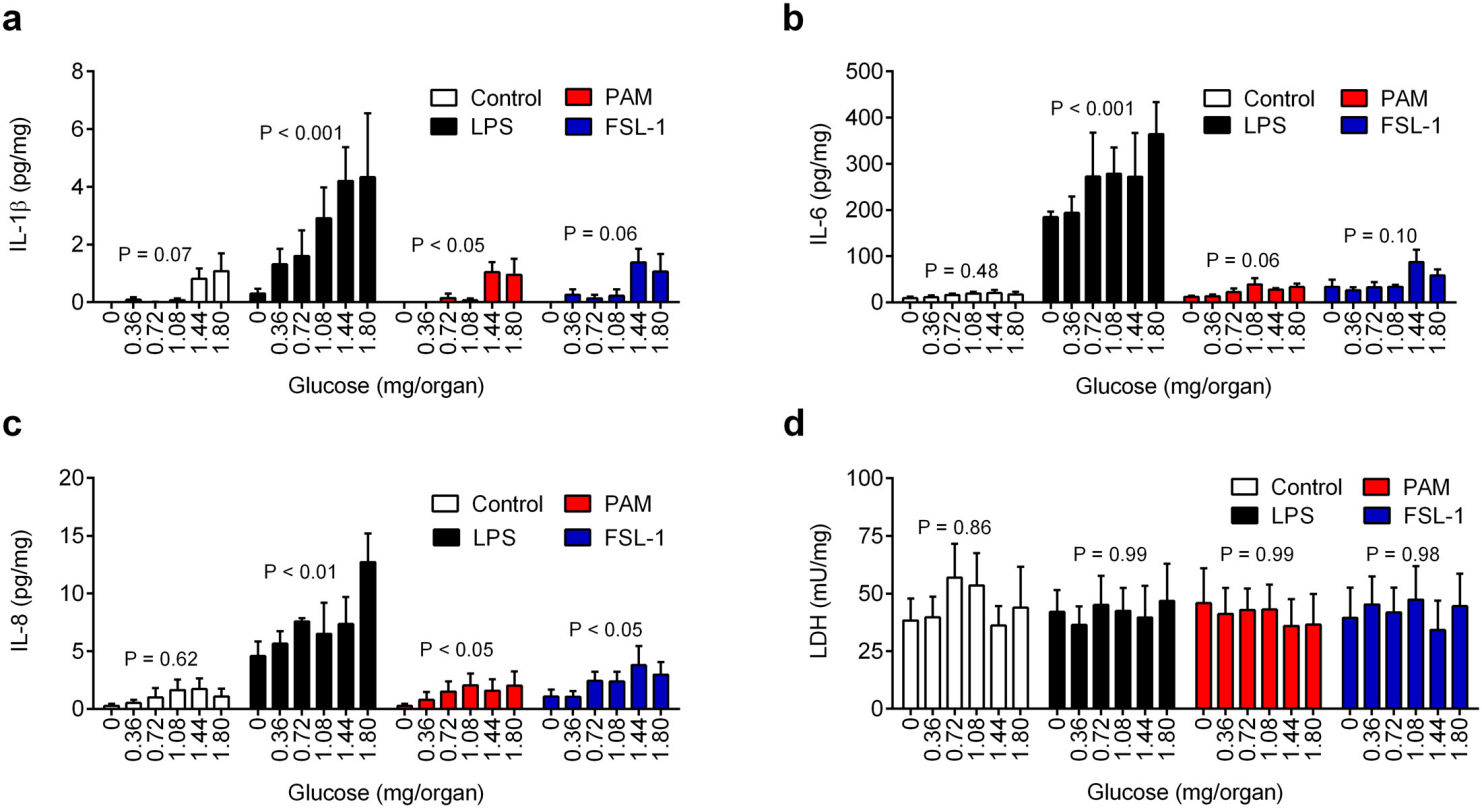
Reduced glucose availability limits inflammatory responses to pathogen-associated molecular patterns. (a-d) *Ex vivo* organ cultures of endometrium were cultured for 24 h in media containing 0 to 1.8 mg/organ glucose, with control vehicle or challenged with media containing 100 ng/ml LPS, PAM or FSL-1. At the end of the experiment organ weights were recorded and the accumulation of IL-1β (a), IL-6 (b), IL-8 (c) and LDH (d) measured in supernatants. Data are presented as mean + SEM concentration per mg tissue from 4 independent experiments, and analyzed by ANOVA, with P values reported above each treatment.

To examine the impact of glucose deprivation on the innate immune response, 8-mm diameter organ cultures of bovine endometrium were placed in 12-well plates for 24 h in media containing a range of amounts of glucose (0 to 1.8 mg/organ), with control vehicle or LPS from *E*. *coli* (100 ng/ml, ligand for TLR4), Pam3CysSerLys4 (PAM, 100 ng/ml, lipopeptide ligand for TLR2/TLR1), or fibroblast-stimulating ligand-1 (FSL-1, 100 ng/ml, lipopeptide ligand for TLR2/TLR6). The maximal 1.8 mg/organ glucose was generated by supplying each culture well with 2 ml of medium containing 5 mM glucose. Although the precise availability of glucose in the postpartum endometrium is unknown, and will likely be less than that in peripheral plasma, the highest amount of glucose supplied was selected because the concentration of glucose in peripheral plasma is ~ 4 mM in normal animals [6]. The amounts of glucose were expressed as mg/organ to focus on glucose availability rather than glucose concentrations, which are more relevant to studies of circulating blood cells than to tissues. We previously validated that endometrial organ cultures secrete IL-1β, IL-6 and IL-8 after 24 h challenge with LPS [28, 29]. However, limiting glucose availability reduced the accumulation of IL-1β, IL-6 and IL-8 in response to LPS by at least 50% (Fig 1a–1c, n = 4 animals). Compared with maximum glucose availability, the effect of limiting glucose on IL-1β, IL-6 and IL-8 responses to LPS was first evident at 0.72, 0.36 and 0.72 mg/organ glucose, respectively (P < 0.05; ANOVA with Dunnett’s pairwise multiple comparison test). Limiting glucose availability also tended to reduce the IL-1β, IL-6 and IL-8 responses to PAM and FSL-1 (Fig 1a–1c). One concern was that glucose deprivation and/or pathogen-associated molecular patterns could indirectly limit the accumulation of inflammatory mediators by reducing cell viability. Cell viability was evaluated by measuring the concentration of lactate dehydrogenase (LDH) in organ supernatants because LDH leaks from damaged cells [28, 29]. However, the concentration of LDH in supernatants did not differ significantly amongst challenges (Fig 1d). Subsequent experiments used LPS because inflammatory responses to LPS were the most evident amongst the pathogen-associated molecular patterns.

**Fig 2:**
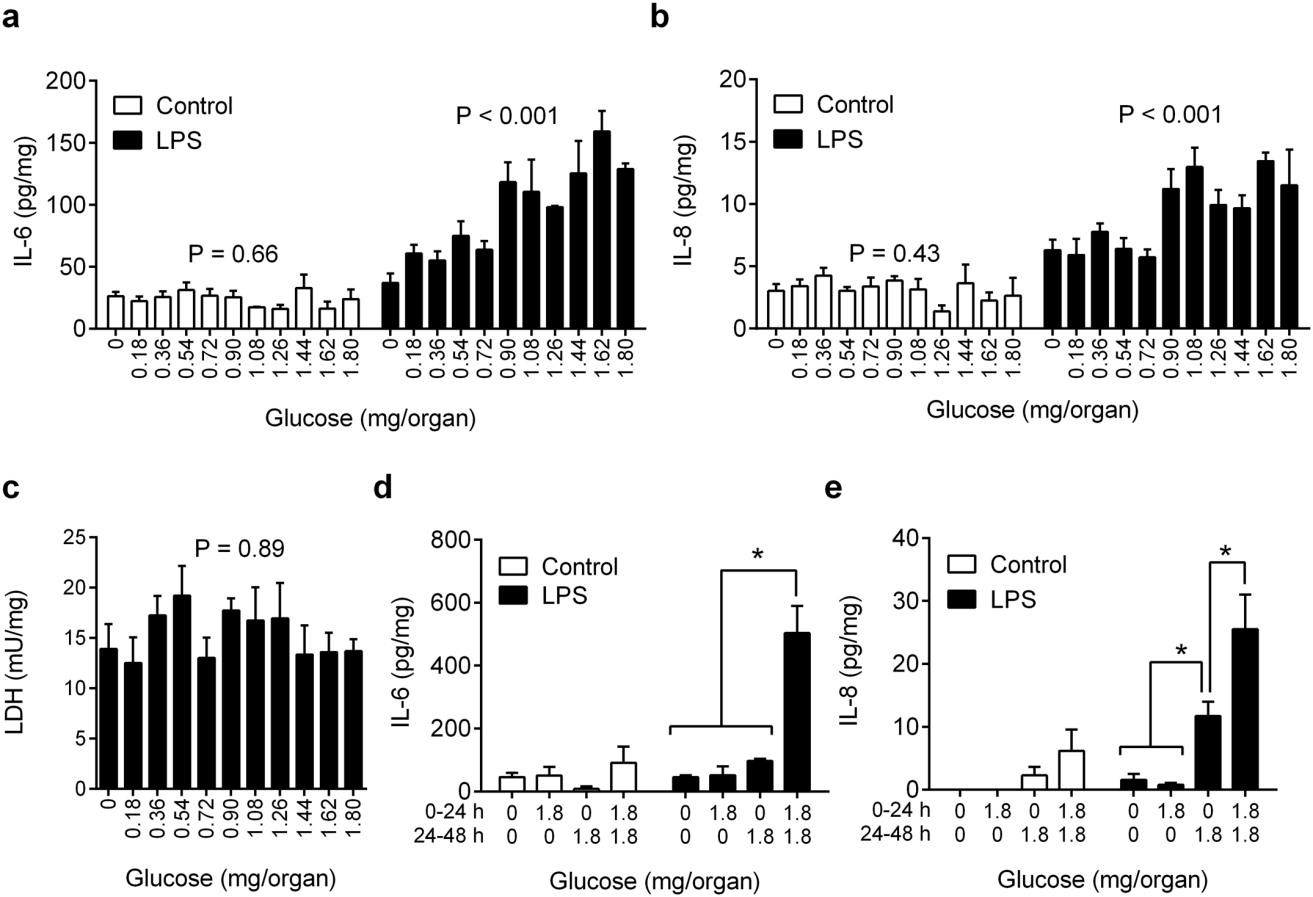
Glucose availability modulates inflammation in endometrial tissue. (a-c) *Ex vivo* organ cultures of endometrium were cultured for 24 h in media containing 0 to 1.8 mg/organ glucose at double the concentration of Fig 1 (maximum 10 mM vs 5 mM in Fig 1), with control vehicle or 100 ng/ml LPS. At the end of the experiment organ weights were recorded and the accumulation of IL-6 (a), IL-8 (b) and LDH (c) measured in supernatants. Data are presented as mean + SEM concentration per mg tissue from 7 independent experiments, and analyzed by ANOVA, with P values reported. (d-e) Endometrial organ cultures were placed in media containing 0 or 1.8 mg/organ glucose for 24 h, and then replaced with media containing 0 or 1.8 mg/organ glucose with control vehicle or 100 ng/ml LPS for a further 24 h. At the end of the experiment organ weights were recorded and the accumulation of IL-6 (d) and IL-8 (e) measured in supernatants. Data are presented as mean + SEM concentration per mg tissue from 4 independent experiments, and analyzed by ANOVA using Bonferroni multiple comparisons test; * P < 0.05.

We next considered whether the effect of glucose availability on inflammation was dependent on the absolute amount of glucose available to the tissues or the concentration of glucose in the culture media. Endometrial organ cultures were placed in 24-well plates for 24 h in the same range of amounts of glucose as used in Fig 1a–1d (0 to 1.8 mg glucose per organ) but using half the volume of media per organ (1 ml/well). This approach doubled the concentrations so that the maximum concentration of glucose was 10 mM (Fig 2a–2c), compared with 5 mM in Fig 1a–1d. Limiting glucose availability reduced the accumulation of IL-6 and IL-8 in response to LPS by at least 45% (Fig 2a and 2b; n = 7 animals) but did not significantly affect cell viability, as determined by LDH concentrations (Fig 2c). The concentration of IL-1β was not measured because the 1 ml medium provided insufficient supernatant to conduct a fourth assay. The effect of limiting glucose on IL-6 and IL-8 responses to LPS was first evident at 0.72 mg/organ glucose, compared with maximum glucose availability (P < 0.05; ANOVA with Dunnett’s pairwise multiple comparison test). These data provide evidence that glucose availability, rather than glucose concentration, influenced the tissue inflammatory responses.

We then wondered whether the reduced inflammatory responses to LPS associated with limiting glucose availability might be rescued by re-feeding cells with glucose. So, endometrial organ cultures were placed in 12-well plates using 2 ml glucose-free media or media containing 1.8 mg/organ glucose for 24 h; then the media were replenished with 2 ml fresh glucose-free media or media containing 1.8 mg/organ glucose, with control vehicle or LPS (100 ng/ml) for 24 h. Deficits in glucose in the first or second 24 h period reduced the accumulation of IL-6 and IL-8 by at least 50% (Fig 2d and 2e) compared with cells maintained throughout the experiment in 1.8 mg glucose; although, re-feeding glucose after a period of depletion partially rescued the IL-8 response (Fig 2e).

Taken together, the data from Figs 1 and 2 support the concept that reducing glucose availability to endometrial tissue limits inflammatory responses to pathogen-associated molecular patterns.

### AMPK modulates inflammation

**Fig 3:**
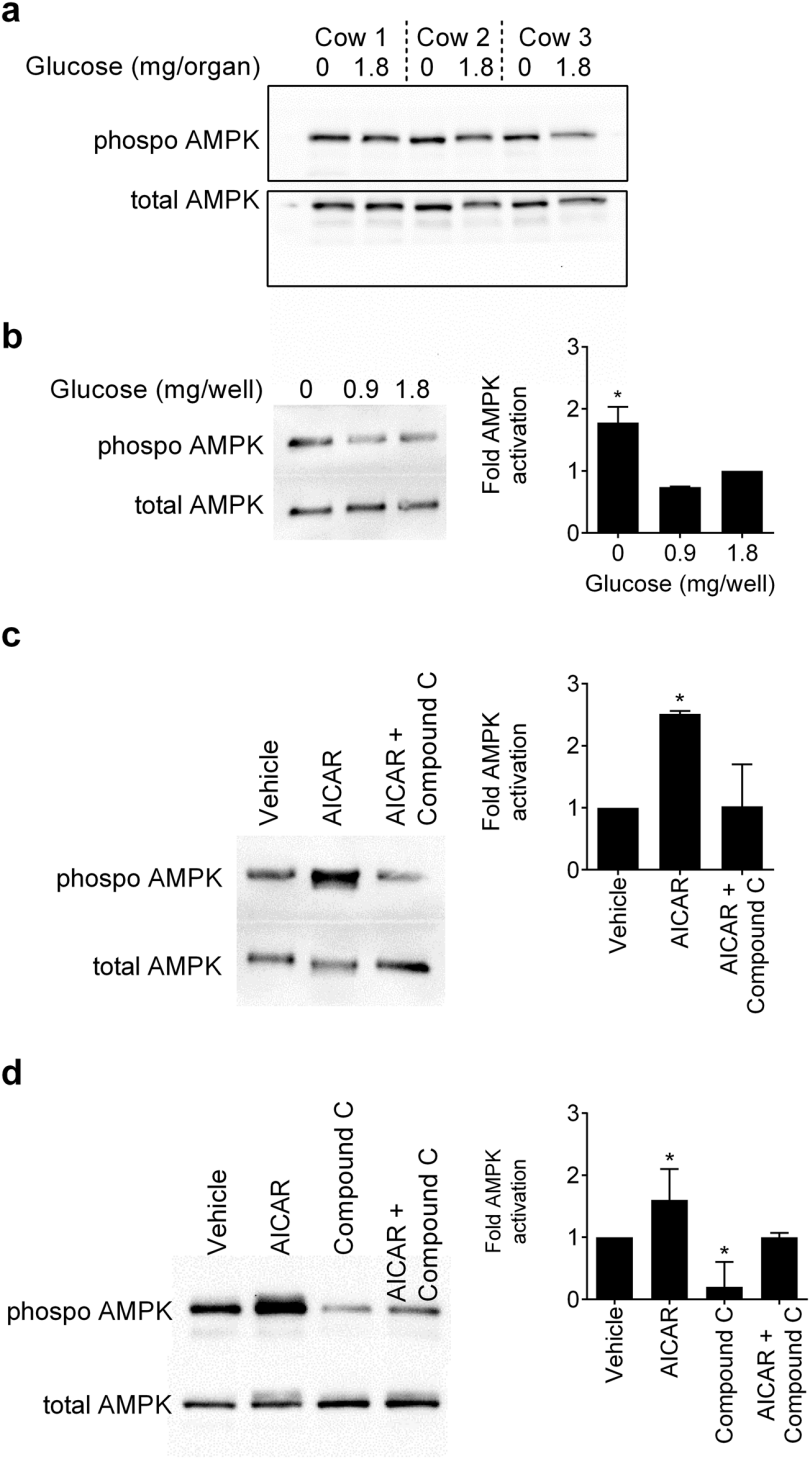
Expression of phosphorylated AMPK. (a) An image of a Western blot from 3 animals where *ex vivo* organ cultures of endometrium were cultured for 4 h in medium containing 0 or 1.8 mg/organ glucose, the samples homogenized and Western blotting performed to evaluate phosphorylated AMPK, with total AMPK used as a loading control. (b) Endometrial stromal cells were cultured for 4 h in medium with 0, 0.9 or 1.8 mg/well glucose, or (c) cultured for 2 h in medium with vehicle, 1 mM AICAR, or AICAR with 50 μM Compound C. (d) Epithelial cells were cultured for 2 h in medium with vehicle, 1 mM AICAR, 50 μM Compound C, or AICAR with Compound C. (b-d) The cells were collected and immunoblotted for phosphorylated AMPK, with protein loading evaluated and normalised to expression of total AMPK. The left panel shows a representative blot from three independent experiments and the right panel mean + SEM of densitometric analysis of phosphorylated AMPK normalized to total AMPK protein, expressed as fold activation of 1.8 mg/organ glucose (b) or vehicle (c, d), and analyzed by Mann-Whitney U test, * P < 0.05.

The next question was to consider how energy deficits, such as limiting glucose availability, might be integrated with innate immunity. The most fundamental intracellular sensor of metabolic energy is AMPK, which is phosphorylated when there is an energy deficit [11]. Using endometrial organ cultures in glucose-free medium or medium containing 1.8 mg/organ glucose for 4 h, the expression of phosphorylated AMPKα was examined by immunoblotting, with protein loading evaluated and normalised to expression of total AMPK (Fig 3a, n = 3 animals). Whilst some endometrial organ cultures had more abundant phosphorylated AMPK with glucose deprivation, the changes in protein abundance were somewhat inconsistent. So, to further examine AMPK phosphorylation we chose the most abundant cells in endometrial tissue, and isolated pure populations of endometrial stromal and epithelial cells [26]. The culture of endometrial stromal cells in the absence of glucose increased the phosphorylation of AMPK compared with 0.9 mg or 1.8 mg glucose (Fig 3b). Furthermore, the phosphorylation of AMPK was increased when stromal cells were treated with 1 mM AICAR (Fig 3c), which is an activator of AMPK [31]. As the level of constitutive AMPK phosphorylation was low in stromal cells, we confirmed that phosphorylation of AMPK was prevented by 50 μM Compound C (Fig 3c), which is a competitive inhibitor of AMPK [32]. With epithelial cells, AICAR increased the phosphorylation of AMPK, and Compound C reduced the constitutive and the AICAR-induced phosphorylation of AMPK (Fig 3d).

**Fig 4:**
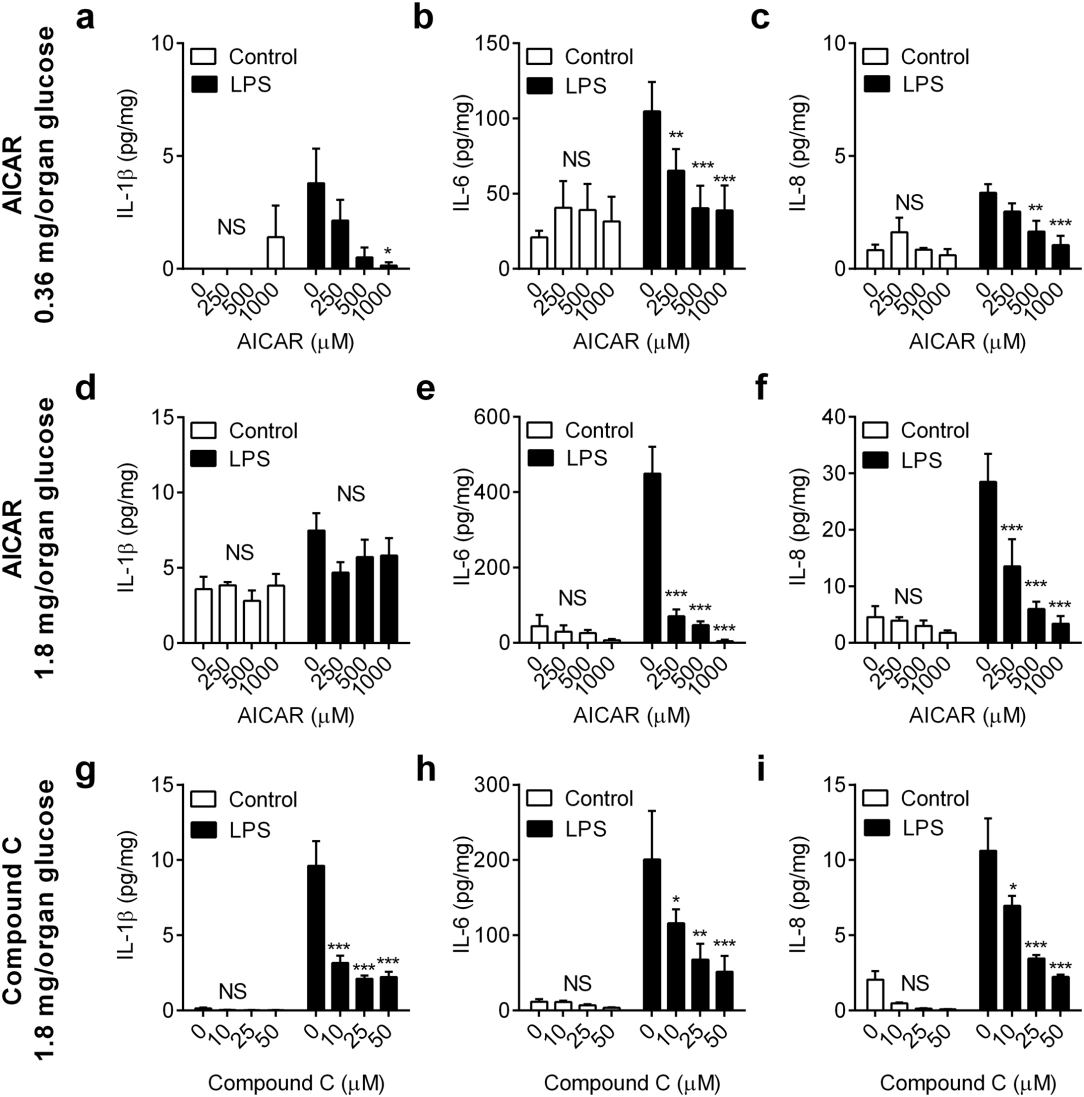
Manipulation of AMPK activity regulates inflammation in endometrial tissue. *Ex vivo* organ cultures of endometrium were cultured for 24 h in medium containing 0.36 mg/organ glucose (a-c) or 1.8 mg/organ glucose (d-f) with vehicle (0) or AICAR (250, 500, 1000 μM) to activate AMPK, or (g-i) in medium containing 1.8 mg/organ glucose with vehicle (0) or Compound C (10, 25, 50 μM) to inhibit AMPK. Media was then aspirated and replenished with fresh medium containing a corresponding concentration of glucose and AICAR or Compound C, and challenged with control vehicle or 100 ng/ml LPS for a further 24 h. At the end of the experiment, organ weights were recorded, and the accumulation of IL-1β (a, d, g), IL-6 (b, e, h) and IL-8 (c, f, i) was measured in supernatants. Data are presented as mean concentration per mg tissue + SEM from 4 independent experiments, and analyzed by ANOVA using Dunnett’s multiple comparisons test to compare with vehicle (0), within each treatment group; * P < 0.05, ** P < 0.01, *** P < 0.001, NS = ANOVA not significant.

To examine the impact of AMPK activation on innate immunity, endometrial organ cultures were placed in medium containing 0.36 mg/organ glucose for 24 h with vehicle or the AMPK activator AICAR (250, 500 or 1000 μM); the medium was then aspirated and explants were challenged for 24 h with medium containing control vehicle or 100 ng/ml LPS, as well as the corresponding concentration of AICAR. Treatment with AICAR reduced the LPS-stimulated accumulation of IL-1β, IL-6 and IL-8 by > 50%, and in a concentration-dependent manner (Fig 4a–4c, n = 4 animals). As well as the action of AICAR, limiting glucose to 0.36 mg/organ might also activate AMPK. So, an additional experiment was performed using AICAR in medium containing 1.8 mg/organ glucose for 24 h, followed by challenge with control vehicle or LPS, as well as the corresponding concentration of AICAR. In response to LPS, the accumulation of IL-6 and IL-8, although not IL-1β, was reduced by > 50% (Fig 4d–4f, n = 4). As 24 h treatment with AICAR might be considered rather protracted, the experiments were also performed using 6 h treatment with AICAR prior to the 24 h LPS challenge. As with 24 h treatment, the 6 h treatment with AICAR reduced the accumulation of IL-6 and IL-8 by > 50% (P < 0.05), but not IL-β, in supernatants of endometrial organ cultures challenged with LPS in media containing 0.36 mg/organ glucose (a-c in S1 Fig) or 1.8 mg/organ glucose (d-f in S1 Fig). There was no significant effect on cell viability when endometrial tissues were cultured with AICAR, as determined by the accumulation of LDH in supernatants (vehicle vs. 1000 μM AICAR, 26.4 ± 2.7 vs. 26.0 ± 2.0 mU/mg, mean ± SEM; P = 0.88, independent t-test, n = 4).

As inflammatory responses to LPS were reduced by at least 50% in media deficient in glucose (Fig 1a–1c) or following treatment with AICAR (Fig 4a–4c), we explored whether inhibition of constitutively active AMPK with Compound C might increase inflammation. Endometrial organ cultures were placed in medium containing 1.8 mg/organ glucose for 24 h with vehicle or Compound C (10, 25 or 50 μM); the medium was then aspirated and explants were challenged for 24 h with medium containing control vehicle or 100 ng/ml LPS, as well as the corresponding concentration of Compound C. However, to our surprise, Compound C reduced the accumulation of IL-1β, IL-6 and IL-8 by > 50%, and in a concentration-dependent manner (Fig 4g–4i). Similarly, a shorter 6 h treatment with Compound C also reduced the inflammatory responses to LPS by > 50% (g-i in S1 Fig). There was no significant effect on cell viability, as determined by the accumulation of LDH in supernatants, when endometrial tissues were cultured with Compound C (vehicle vs. 50 μM Compound C, 313 ± 25 vs. 318 ± 65 mU/ml; P = 0.72, t-test, n = 4).

Taken together, the data from Figs 3 and 4 provide evidence that AMPK is an important molecular control point for metabolic interaction with innate immunity, with reduced or increased AMPK activation limiting endometrial inflammatory responses to LPS.

### mTOR and inflammation

**Fig 5:**
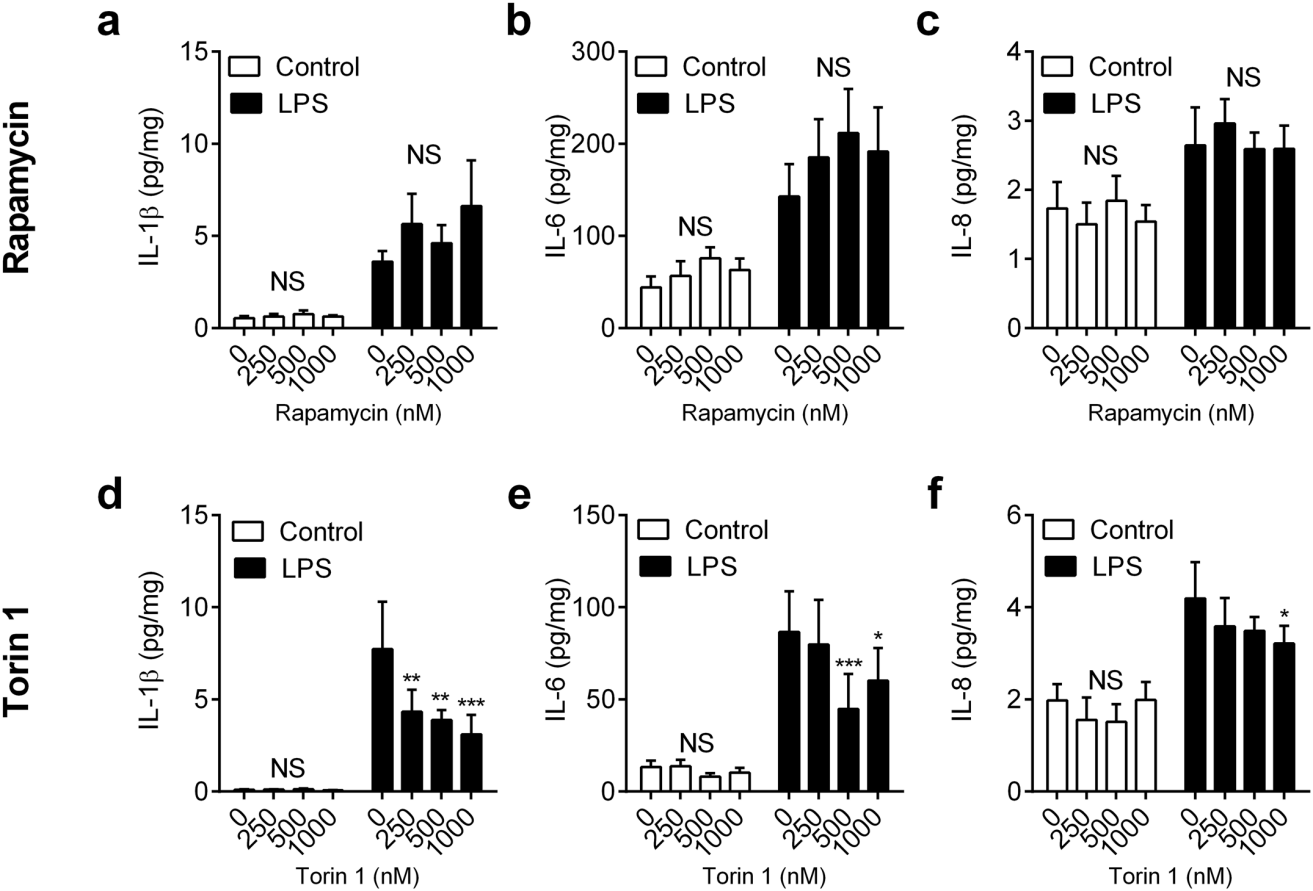
Impact of inhibiting mTOR on inflammation in endometrial tissue. *Ex vivo* organ cultures of endometrium were cultured for 24 h in medium containing 1.8 mg/organ glucose with vehicle (0), or rapamycin (a-c: 250, 500, 1000 nM) or Torin 1 (d-f: 250, 500, 1000 nM). Media was then aspirated and replenished with fresh medium containing a corresponding concentration of glucose and rapamycin or Torin 1, and challenged with control vehicle or 100 ng/ml LPS for a further 24 h. At the end of the experiment, organ weights were recorded, and the accumulation of IL-1β (a, d), IL-6 (b, e) and IL-8 (c, f) was measured in supernatants. Data are presented as mean concentration per mg tissue + SEM from 4 independent experiments, and analyzed by ANOVA using Dunnett’s multiple comparisons test to compare with vehicle (0), within each treatment group; * P < 0.05, ** P < 0.01, *** P < 0.001, NS = ANOVA not significant.

Unlike AMPK which specifically senses the AMP:ATP ratio, mTOR integrates multiple indicators of nutrient availability, including signals from amino acids and IGF-1, as well as AMPK [12, 16]. There are two mTOR complexes (mTORC1 and mTORC2), and mTORC1 is down regulated when faced with a nutrient deficit, although little is known about the regulation of mTORC2 [12, 16]. To examine the role of mTOR in modulation of innate immunity, we used the mTORC1 inhibitor rapamycin, and the dual mTORC1 and mTORC2 inhibitor Torin 1 [33, 34]. Endometrial organ cultures were placed in medium containing 1.8 mg/organ glucose with vehicle, or a range of concentrations of rapamycin (250, 500 or 1000 nM), or Torin 1 (250, 500 or 1000 nM) for 24 h; the medium was then aspirated and explants were challenged for 24 h with medium containing control vehicle or 100 ng/ml LPS, as well as the corresponding concentration of rapamycin or Torin 1. The inhibitors did not significantly affect endometrial tissue viability, as determined by LDH accumulation (all values within ± 13% of control; P = 0.57, ANOVA, n = 4 animals). Furthermore, although LPS stimulated the accumulation of more IL-1β, IL-6 and IL-8 in supernatants when compared with control (P < 0.01, ANOVA), as expected, the inflammatory response was not significantly modified by treatment with rapamycin (Fig 5a–5c, P = 0.20, ANOVA, n = 4). Similarly, there was no significant effect on inflammatory mediator accumulation with a shorter 6 h rapamycin treatment prior to challenge with LPS for 24 h (a-c in S2 Fig). On the other hand, there was a modest reduction in LPS-stimulated IL-1β, IL-6 and IL-8 accumulation following 24 h treatment with Torin 1 (Fig 5d–5f, P < 0.05, ANOVA, n = 4). However, a shorter 6 h treatment with Torin 1 did not significantly affect the accumulation of IL-1β or IL-8 in response to LPS, although there was a modest reduction in IL-6 secretion (d-f in S2 Fig). Together these data provide evidence that mTORC1 is not a major regulator of inflammatory responses to LPS, although the limited effect of Torin 1 might imply a role for mTORC2 in modulating endometrial innate immunity.

### IGF-1 and inflammation

**Fig 6:**
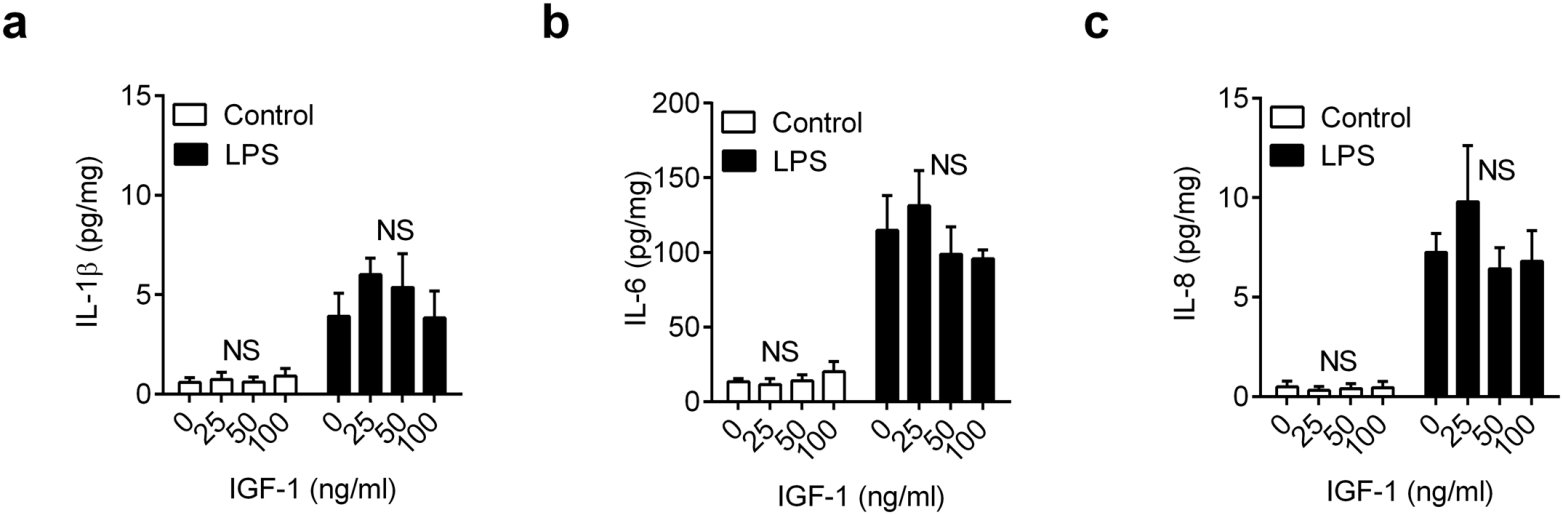
IGF-1 does not modulate inflammation in endometrial tissue. *Ex vivo* organ cultures of endometrium were cultured for 24 h in 1.8 mg/organ glucose-containing medium with vehicle (0) or IGF-1 (25, 50, 100 ng/ml). Media was then aspirated and replenished with fresh medium containing a corresponding concentration of glucose and IGF-1, and challenged with control vehicle or 100 ng/ml LPS for a further 24 h. At the end of the experiment, organ weights were recorded, and the accumulation of IL-1β (a), IL-6 (b) and IL-8 (c) measured in supernatants. Data are presented as mean concentration per mg tissue + SEM from 4 independent experiments, and analyzed by ANOVA, NS = not significant.

Insulin-like growth factor-1 regulates cellular growth and metabolism [35, 36]. Compared with normal animals, postpartum animals under metabolic stress have lower plasma concentrations of IGF-1, typically < 50 ng/ml [37]. Thus, we examined whether additional IGF-1 might increase inflammatory responses to pathogen-associated molecular patterns. Endometrial organ cultures were placed in medium containing 1.8 mg/organ glucose with vehicle or a range of concentrations of IGF-1 (25, 50 or 100 ng/ml) for 24 h; the medium was then aspirated and explants were challenged for 24 h with medium containing control vehicle or 100 ng/ml LPS, as well as the corresponding concentration of IGF-1. The accumulation of IL-1β, IL-6 and IL-8 in supernatants following LPS challenge was not significantly modulated by IGF-1 treatment (Fig 6a–6c, P > 0.41, ANOVA, n = 4 animals); and IGF-1 did not significantly affect cell viability, as determined by measuring concentrations of LDH in supernatants (all values within ± 10% of control, P = 0.56, ANOVA,). A shorter 6 h treatment with IGF-1, prior to 24 h challenge with LPS, yielded similar results with no significant effect on IL-1β, IL-6 or IL-8 accumulation (a-c in S3 Fig). These data provide evidence that IGF-1 was not an important regulator of endometrial inflammatory responses to LPS.

### Responses to LPS are energetically expensive

**Fig 7:**
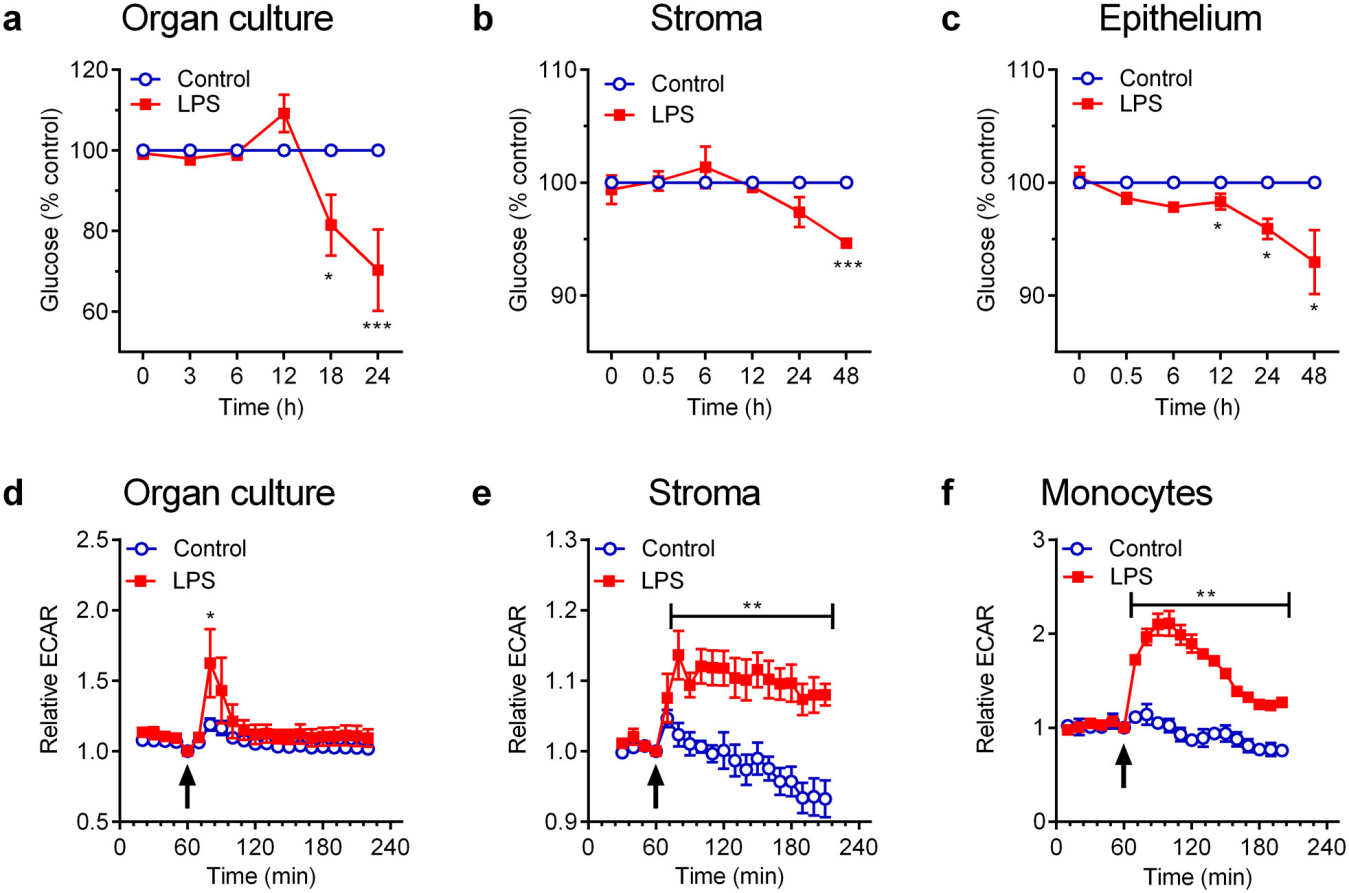
LPS induces metabolic stress in endometrial tissue and cells. (a-c) Endometrial organ cultures (a), stromal cells (b) or epithelial cells (c) were cultured in glucose-containing culture medium with control vehicle (○) or 100 ng/ml LPS (■), and the amounts of glucose in the supernatants was measured at the indicated times. Data are presented as mean ± SEM percent of control, from 4 independent experiments, and analyzed by ANOVA using Sidak's multiple comparisons test; values differ from control, * P < 0.05, *** P < 0.001. (d-f) Endometrial organ cultures (d), stromal cells (e) or monocytes (f) were cultured for 60 min and then remained as unstimulated controls or were challenged (arrow) with 1 μg/ml LPS. The extracellular acidification rate (ECAR) was measured every 10–12 min using an XF-24 Extracellular Flux Analyzer. Data are expressed relative to ECAR at the time of challenge, presented as mean ± SEM from 4 independent experiments, and analyzed by ANOVA using Sidak's multiple comparisons test; values differ from control, * P < 0.05, ** P < 0.01.

Having established that glucose deprivation (Fig 1) and manipulation of AMPK (Fig 4) had the greatest impact on inflammation, the final step was to examine if endometrial responses to LPS were energetically expensive, which might exacerbate the impact of metabolic stress on innate immunity in tissues. Endometrial organ cultures were placed in 2 ml/well control medium or in medium containing 100 ng/ml LPS in 12-well plates, and glucose concentrations measured over 24 h. In addition, because we were aware that tissue contains many more cells than a culture monolayer, the experiments were also repeated using primary endometrial stromal and epithelial cells, using 1 ml/well in 24-well plates, and glucose concentrations measured over 48 h. Data were expressed as the total glucose in each well and, as expected, glucose was depleted from control cell culture medium by organ cultures (0 vs 24 h, 1.77 ± 0.02 vs. 0.95 ± 0.06 mg/well, P < 0.001, t-test, n = 4 animals). Similarly, glucose was depleted from control medium by epithelial cell cultures (0 vs 48 h, 1.93 ± 0.01 vs. 1.57 ± 0.07 mg/well, P < 0.001, t-test, n = 4) and by stromal cell cultures (0 vs 48 h, 1.84 ± 0.03 vs. 1.52 ± 0.11 mg/well, P < 0.001, t-test, n = 4). However, when organ or cell cultures were challenged with LPS, there was an increased rate of depletion of glucose from the culture media, compared with control (Fig 7a–7c, P < 0.05, ANOVA, n = 4).

As LPS stimulated glucose consumption, we used an Extracellular Flux Analyzer (Seahorse Bioscience) to examine whether LPS induced the Warburg effect. The extracellular acidification rate (ECAR) was measured every 10 to 12 min over a 4 h period, as an indicator of lactate production, when endometrial organ cultures were challenged with control vehicle or LPS in medium containing 1.19 mg/organ glucose. As there is limited data on ECAR, endometrial stromal cells and peripheral blood monocytes were employed as comparators [38]. Prior to challenge with LPS, the ECAR was 15.3 ± 7.3, 20.3 ± 2.3 and 13.7 ± 1.6 mpH/min (mean ± SEM) for endometrium, stromal cells and monocytes, respectively. However, the ECAR was increased in response to LPS for endometrial organ cultures (Fig 7d, P < 0.05, ANOVA, n = 3 animals), stromal cells (Fig 7e, P < 0.001, ANOVA, n = 3) and monocytes (Fig 7f, P < 0.001, ANOVA, n = 3). Together these data support the concept that the endometrium uses glucose as a carbon source, and that LPS further stimulates the consumption of glucose.

## Discussion

The rationale for the present studies was that metabolic energy deficits are associated with inadequate immune responses and uterine disease [6, 9, 10]. We reasoned that the regulation of metabolic energy and inflammatory responses to pathogens both aim to achieve tissue homeostasis, and are likely to be integrated [1, 11, 22]. Here we provide evidence that interactions between metabolism and innate immunity depend on glucose availability and AMPK activity, which are both fundamental to maintaining energy homeostasis in tissues. Glucose deprivation reduced endometrial organ culture inflammatory responses to pathogen-associated molecular patterns, and activation of AMPK reduced IL-6 and IL-8 secretion in response to LPS, even if there was sufficient glucose available. Surprisingly, inhibition of AMPK activity also perturbed inflammation, implying that a constitutive level of AMPK activity is important for optimal immunity. In contrast, the more generalized regulators of metabolism, mTOR and IGF-1, had little effect on the innate immune response. Finally, the inflammatory response to LPS was energetically expensive and induced the Warburg effect. Taken together, these data provide evidence that glucose availability and AMPK impact innate immunity in the bovine endometrium.

The most fundamental source of cellular energy is ATP, derived mainly from metabolism of glucose through glycolysis and oxidative phosphorylation in the Krebs cycle [11, 17]. To examine innate immune responses to pathogen-associated molecular patterns we exploited *ex vivo* organ cultures of bovine endometrium because they mimic *in vivo* inflammatory responses to bacteria [28, 29]. In the present study, limiting glucose availability reduced IL-1β, IL-6 and IL-8 accumulation from endometrial tissue in response to LPS, although there were more modest effects on the inflammatory responses to lipopeptides. Notably, the effect of glucose on inflammation was determined by glucose availability, rather than the concentration of glucose in the medium. Furthermore, inflammation was impaired by any deficit in glucose availability, prior to or during LPS challenge, and re-feeding endometrium with glucose only partially rescued the effect.

The rate of consumption of glucose by control endometrial organ cultures in the present study was about 25 ng/mg/min, which is similar to previous observations [39]. However, deficits in glucose availability in tissues, such as during the postpartum period, are exacerbated by increased glucose consumption. In the present study, there was increased consumption of glucose when endometrial tissue or cells were challenged with LPS. Furthermore, LPS induced the Warburg effect in the endometrial tissue, as determined by increased ECAR. These observations are similar to previous work with murine and human dendritic cells and macrophages, where LPS also induced the Warburg effect [19, 38, 40]. Such metabolic adaptations to LPS are likely part of the integrated innate immune response during bacterial infections [2, 22]. However, beyond adaptation, metabolic stress clearly perturbed inflammation in the present study, and limits immunity *in vivo* [6]. This perturbation of inflammatory responses is important because insufficiently rapid or robust inflammatory responses leads to persistent infection and/or chronic inflammation [22], which is a cardinal sign of endometritis in cattle [3]. Metabolic stress compromising defence against microbes is also evident in other species. Even bumblebees are more likely to die when challenged with LPS, if they are starved of sugar [41].

In the present study, the role of AMPK was explored to seek mechanistic links between energy metabolism and innate immunity. We first confirmed that glucose deprivation increased phosphorylation of AMPK, as might be expected when there is metabolic stress [11]. More importantly, the inflammatory response to LPS was reduced by activation of AMPK, mimicking an energy deficit. This anti-inflammatory effect of AMPK activation has been suggested to be associated with increased catabolism and inhibition of mTOR [2]. Our most surprising observation was that competitive inhibition of AMPK activity by Compound C reduced inflammation, rather than enhancing inflammation as might be predicted. These data support previous observations using human THP-1 macrophage-like cells, where Compound C reduced IL-1β, IL-6 and TNFα secretion in response to LPS [42]. However, there is a contradictory report where Compound C increased *IL1B*, *IL6* and *TNF* gene expression in response to LPS in murine macrophages [43]. The reason for discrepancies between studies is not clear, but abundance of mRNA may not translate to secretion of protein when metabolism is compromised. On balance, it appears that a constitutive level of AMPK activity may be required for optimal innate immunity.

The next step was to consider if the more generalized regulators of tissue metabolism, mTOR or IGF-1, might modulate inflammatory responses to LPS in the endometrium. Inhibiting the principal sensor of cell nutrients mTORC1 with rapamycin did not influence the response to LPS. However, mTORC2 may have a role because Torin 1 partially inhibited inflammation. Differential roles for mTORC1 and mTORC2 have also been identified in T-cell differentiation [44]. Little is known about the mechanisms of action of mTORC2, although there is evidence that mTORC2 modulates protein kinase B (also known as Akt), protein kinase C and S6 kinase, and IGF-1 is an upstream regulator of mTORC2 [12]. As IGF-1 concentrations are reduced in the peripheral plasma after parturition, and IGF binding proteins are differentially regulated in the postpartum uterus, it was thought that IGF-1 may be directly responsible for mediating the effects of negative energy balance on uterine health [5, 6, 45]. However, the present study counters this idea, as exogenous IGF-1 did not modulate the inflammatory responses to LPS in the endometrium.

The postpartum endometrium likely faces the conflict of impaired innate immunity because of reduced availability of glucose, and increased cellular metabolism during the inflammatory response to bacteria. Indeed, the endometrium is likely further metabolically compromised *in vivo* by demands to use glucose for energy and as a carbon substrate to meet the needs of the extensive tissue repair and regeneration that occurs in the postpartum endometrium [46]. These additional metabolic stresses may explain why endometrial defence is compromised during postpartum negative energy balance whereas innate immunity in the mammary gland is not compromised during induced negative energy balance [6, 30].

Cellular energy metabolism depends on the transport and utilization of a multitude of carbon substrates [1, 11, 12]. Glucose transporters regulate the movement of glucose into cells, and some are regulated by ovarian steroids and insulin in murine and human endometrium [13]. However, the stage of ovarian cycle does not affect glucose transporter expression in the bovine endometrium [14]; and, ovarian steroids do not affect endometrial inflammatory responses to LPS [29]. Similarly, insulin and IGF-1 are not required by *ex vivo* endometrial tissue for physiological function, as determined by prostaglandin secretion in response to oxytocin, or inflammatory responses to pathogen-associated molecular patterns [28, 47]. Ovarian steroids and IGF-1 may have roles in other processes that promote uterine health. The IGF-1 and insulin systems are important for pospartum endometrial repair, probably by regulating matrix metalloproteinases and cell proliferation [37]. When glucose supply is limiting, energy requirements can often be met through metabolism of amino acids, lipids and long chain fatty acids. Glutaminolysis generates substrates such as succinate for the Krebs cycle. Fatty acids yield acetyl CoA for the Krebs cycle, and for the mevalonate pathway, which converts acetyl CoA to squalene for cholesterol and steroid synthesis. These metabolic pathways are subject to multiple control systems, including AMPK and mTOR, and their interactions with innate immunity are only just emerging [2, 11, 12]. For example, we recently found that bovine endometrial cell inflammatory responses to LPS are blunted by inhibition of the mevalonate pathway or addition of isoprenoids [48]. Future work may explore whether the AMPK control point is generalized across the component cells of the endometrium, or is specific to certain cells. A multi-dimensional approach might be used to explore how immunity and tissue repair are impacted by contemporaneous manipulation of AMPK and carbon substrates, such as glucose, glutamine and long chain fatty acids. Indeed, in the present study, it was interesting that activation of AMPK only reduced IL-1β when glucose was limited, whereas the effects on IL-6 and IL-8 were independent of glucose. This differential integration of metabolism and immunity is reminiscent of observations with murine macrophages, where succinate regulates IL-1β, but not IL-6, secretion in response to LPS [19].

In conclusion, here we provide evidence that metabolic energy stress in the endometrium perturbs the inflammatory response to pathogen-associated molecular patterns. In particular, glucose availability and the intracellular sensor of energy, AMPK, mechanistically linked metabolism and immunity in the endometrium.

## Materials and Methods

### Organ and cell culture

Uteri with no gross evidence of genital disease or microbial infections were collected from cattle (n = 68 animals), after they were slaughtered and processed as part of the normal work of an abattoir, as described previously [26–29]. *Ex vivo* organ cultures of bovine endometrium were generated using 8 mm diameter biopsy punches, as described previously [28, 29]. We previously demonstrated that tissue architecture was maintained within the organ culture period, that there was no animal breed effect on inflammatory responses to LPS, and that there was no significant difference in cell viability between control and LPS challenge of endometrial organ cultures [28, 29]. Organ cultures were placed in 12-well plates (TPP, Trasadingen, Switzerland) in 2 ml/well culture medium (RPMI-1640 medium cataologue number R8758, 50 IU/ml of penicillin, 50 μg/ml of streptomycin and 2.5 μg/ml of amphotericin B, Sigma, Gillingham, Dorset, UK), except where indicated in *Results* when 1 ml/well was used in 24-well plates (TPP).

Endometrial stromal and epithelial cell populations were isolated, and the absence of immune cell contamination confirmed as described previously [26, 27]. Epithelial and stromal cells were plated at 1.5 × 10^5^ cells/ml, unless stated otherwise, in 24-well plates using 1 ml/well culture medium (RPMI-1640 medium cataologue number R8758, 50 IU/ml of penicillin, 50 μg/ml of streptomycin and 2.5 μg/ml of amphotericin B, Sigma; with 10% foetal bovine serum, Biosera, Uckfield, East Sussex, UK). Peripheral blood monocytes were isolated as described previously [49]. Monocytes were plated at 6 x 10^4^ cells/ml in culture medium (RPMI-1640 medium cataologue number R8758, 50 IU/ml of penicillin, 50 μg/ml of streptomycin and 2.5 μg/ml of amphotericin B, Sigma; with 10% foetal bovine serum, Biosera) in 24-well plates. Organ and cell cultures were incubated at 37°C in a humidified atmosphere of air with 5% CO2.

### Impact of metabolism on inflammation

Endometrial organ cultures were treated with RPMI media containing a range of amounts of glucose, generated by using combinations of RPMI glucose-free medium (RPMI 1640 no glucose catalogue number 11879, Thermo Scientific, Cramlington, UK) and RPMI medium containing 11 mM glucose (RPMI-1640 medium cataologue number R8758, Sigma). The final amounts of glucose in media were confirmed using a glucose assay (see below). Organ cultures were challenged with vehicle or ultrapure LPS from *E*. *coli* O111:B4 (100 ng/ml, Invivogen, Toulouse, France), PAM3CSK4 (PAM, 100 ng/ml; Invivogen) or fibroblast-stimulating lipopeptide-1 (FSL-1 (also known as Pam2CGDPKHPKSF), 100 ng/ml; Invivogen). After 24 h, supernatants were collected and stored at -20°C for subsequent measurement of IL-1β, IL-6, IL-8 and LDH, and organ weights were recorded. Each experiment was performed using organ cultures from at least four independent animals, and each treatment was replicated twice.

To explore the role of cellular signaling pathways in the innate immune response, organ cultures were placed in media containing defined concentrations of glucose for 6 h or 24 h, with an AMPK activator (0 to 1000 μM AICAR, 5-amino-4-imidazolecarboxamide ribonucleoside, Tocris, Avonmouth, Bristol, UK), an AMPK inhibitor (0 to 50 μM Compound C, 6-[4-(2-Piperidin-1-yl-ethoxy)-phenyl)]-3-pyridin-4-yl-pyyrazolo[1,5-a] pyrimidine, Millipore, Watford, Herfordshire, UK), mTOR inhibitors (0 to 1000 nM, rapamycin, Merck, Watford, Hertfordshire, UK; 0 to 1000 nM, Torin 1, 1-[4-[4-(1-Oxopropyl)-1-piperazinyl]-3-(trifluoromethyl)phenyl]-9-(3-quinolinyl)-benzo[h]-1,6-naphthyridin-2(1H)-one, Tocris), or IGF-1 (0 to 100 ng/ml recombinant human IGF-1, Sigma) according to the manufacturers’ instructions. Media was then aspirated and replenished with fresh medium containing control vehicle or LPS, as well as the corresponding concentration of the prior treatment with glucose, AICAR, Compound C, rapamycin, Torin 1, or IGF-1. After 24 h, supernatants were collected and stored at -20°C for subsequent measurement of IL-1β, IL-6, IL-8 and LDH, and organ weights were recorded. Each experiment was performed using organ cultures from at least four independent animals, and each treatment was replicated twice.

### ELISA

Concentrations of IL-1β, IL-6, and IL-8 in culture supernatants were measured by ELISA according to the manufacturer’s instructions (Bovine IL-1β Screening Set ESS0027, Bovine IL-6 Screening Set ESS0029, Thermo Scientific; Human CXCL8/IL-8 DuoSet DY208, R&D Systems Europe, Abingdon, UK), as described previously [26–28]. The IL-8 assay has previously been shown to cross-react with bovine IL-8 [50]. The inter-assay and intra-assay coefficients of variation were all < 10%; the limits of detection were 12.5 pg/ml for IL-1β, 75.0 pg/ml for IL-6, and 5.7 pg/ml for IL-8.

### Lactate dehydrogenase assay

Cell damage causes the release of lactate dehydrogenase (LDH) into the surrounding milieu, which is used to evaluate the viability of cells and tissues [28]. Lactate dehydrogenase activity was measured using a Lactate Dehydrogenase Activity Assay Kit (Cambridge Bioscience Ltd, Cambridge, UK), according to the manufacturer’s instructions.

### Immunoblotting

To evaluate whether the abundance of phosphorylated AMPK changes in endometrial organ cultures or stromal cells, they were cultured in medium with 0, 5 or 10 mM glucose. To evaluate the effect of AICAR or Compound C on AMPK phosphorylation, stromal and epithelial cells were treated with AICAR (1 mM) and/or compound C (50 μM) for 2 h. Tissues were homogenized and cells lysed, and then proteins extracted, immunoblotted, and quantified using the Chemidoc XRS system, as described previously [27]. The primary antibodies were for total AMPKα (1:1000 dilution; #5831; Cell Signaling, Danvers, MA, USA) and phosphorylated AMPKα (Thr172) (1:1000 dilution; #2535; Cell Signaling).

### Glucose consumption in response to LPS

To examine glucose consumption, endometrial organ cultures or endometrial cells were treated in RPMI-1640 medium (cataologue number R8758, Sigma) with control vehicle or LPS (100 ng/ml), and supernatants collected periodically to measure the concentration of glucose. The concentration of glucose was measured using a clinical chemistry analyzer (Analox GM7; Analox Instruments, London, UK) according to the manufacturer’s instructions. Briefly, the device was calibrated using a glucose standard before 10 μl samples were measured in duplicate. The intra-assay coefficient of variation was 1.4%.

### Extracellular acidification rate

The ECAR was measured, as an indicator of glycolysis, in real-time using an XF-24 Extracellular Flux Analyzer (Seahorse Bioscience, North Billerica, MA, USA). *Ex vivo* organ cultures of bovine endometrium were generated as described above, but using 3 mm-diameter biopsy punches (Stiefel Laboratories Ltd, High Wycome, Buckinghamshire, UK). The cultures were stabilized in 24-well plates containing 1.8 mg glucose (Sigma) in 2  ml XF Assay Medium (pH 7.4; Seahorse Bioscience, North Billerica, Massachusetts, US) in an incubator at 37°C without carbon dioxide. The organ cultures were then placed in XF24 Islet Capture Microplate (Seahorse Bioscience), covered with an islet capture screen, which allows free perfusion of gases while minimising tissue movement, and washed twice with XF Assay medium. The organ cultures were placed in the analyzer and equilibrated for approximately 60 min in 525 μl/well XF Assay Medium with glucose (1.19 mg/organ final glucose available), prior to injection of 75 μl of XF Assay Medium or 75 μl of XF Assay Medium with LPS (1 μg/ml final concentration). Three animals were analysed per plate with 3 replicates per treatment, and ECAR was measured approximately every 10–12 min. Stromal cells (3.5 x 10^4^ cells/well) or peripheral blood mononuclear cells (6 x 10^4^/well) were cultured in (RPMI-1640 medium cataologue number R8758, 50 IU/ml of penicillin, 50 μg/ml of streptomycin and 2.5 μg/ml of amphotericin B, Sigma; with 10% foetal bovine serum, Biosera) in XF24 Cell Culture Microplates (Seahorse Bioscience). After 24 h, the cells were washed twice with XF Assay medium, placed in the analyzer and equilibrated for approximately 60 min in 525 μl/well XF Assay Medium with glucose (1.19 mg/organ final glucose available), prior to injection of 75 μl of XF Assay Medium or 75 μl of XF Assay Medium with LPS (1 μg/ml final concentration). Three animals were analysed per plate with 3 replicates per treatment, and ECAR was measured approximately every 10–12 min.

### Statistical analysis

Data are presented as arithmetic mean and SEM. Statistical analyses were performed using SPSS 20.0 (SPSS Inc., Chicago, IL, USA), with the animal as the designated statistical unit, and P < 0.05 considered significant. Comparisons were made between treatments, as indicated in *Results*, using Student’s t-test, or GLM multivariate ANOVA with Bonferroni, Sidak's or Dunnett’s post hoc multiple comparison tests for normally distributed data, and Mann-Whitney U test for non-normal data.

## Supporting Information

S1 Fig Manipulation of AMPK activity regulates inflammation in endometrial tissue. *Ex vivo* organ cultures of endometrium were cultured for 6 h in medium containing 0.36 mg/organ glucose with AICAR (a-c: 0, 250, 500, 1000 μM), 1.8 mg/organ glucose with AICAR (d-f: 0, 250, 500, 1000 μM), or 1.8 mg/organ glucose with compound C (g-i: 0, 10, 25, 50 μM). Media was then aspirated and replenished with fresh medium containing a corresponding concentration of AICAR or Compound C, and challenged with control vehicle or 100 ng/ml LPS for a further 24 h. At the end of the experiment, organ weights were recorded, and the accumulation of IL-1β (a, d, g), IL-6 (b, e, h) and IL-8 (c, f, i) was measured in supernatants by ELISA. Data are presented as mean concentration per mg tissue + SEM from 4 independent experiments, and analyzed by ANOVA using Dunnett’s multiple comparisons test to compare with vehicle (0), within each treatment group; * P < 0.05, ** P < 0.01, *** P < 0.001, NS = ANOVA not significant.
(PDF)

S2 Fig Impact of inhibiting mTOR on inflammation in endometrial tissue *Ex vivo* organ cultures of endometrium were cultured in media containing 1.8 mg/organ glucose with (a-c) rapamycin or (d-f) torin-1 (0, 250, 500, 1000 nM) for 6 h followed by medium containing control vehicle or 100 ng/ml LPS for 24 h. At the end of the experiment organ weights were recorded, and the accumulation of IL-1β (a, d), IL-6 (b, e) and IL-8 (c, f) was measured in supernatants. Data are presented as mean concentration per mg tissue + SEM from 4 independent experiments. Data are presented as mean concentration per mg tissue + SEM from 4 independent experiments, and analyzed by ANOVA using Dunnett’s multiple comparisons test to compare with vehicle (0), within each treatment group; * P < 0.05, NS = ANOVA not significant.
(PDF)

S3 Fig IGF-1 does not modulate inflammation in endometrial tissue. *Ex vivo* organ cultures of endometrium were cultured in media containing 1.8 mg/organ glucose with IGF-1 (0, 25, 50, 100 ng/ml) for 6 h, followed by challenge with medium containing control vehicle or 100 ng/ml LPS for 24 h. At the end of the experiment organ weights were recorded and the accumulation of IL-1β (a), IL-6 (b) and IL-8 (c) was measured in supernatants. Data are presented as mean concentration per mg tissue + SEM from 4 independent experiments, and analyzed by ANOVA; NS = not significant.
(PDF)
