# Supplementary material for: Glucose Availability and AMP-Activated Protein Kinase Link Energy Metabolism and Innate Immunity in the Bovine Endometrium
Source: PLoS One. 2016 Mar 14;11(3):e0151416. doi: 10.1371/journal.pone.0151416 (PMC4790959; doi:10.1371/journal.pone.0151416)
Supplement: S1 Fig — Ex vivo organ cultures of endometrium were cultured for 6 h in medium containing 0.36 mg/organ glucose with AICAR (a-c: 0, 250, 500, 1000 μM), 1.8 mg/organ glucose with AICAR (d-f: 0, 250, 500, 1000 μM), or 1.8 mg/organ glucose with compound C (g-i: 0, 10, 25, 50 μM). Media was then aspirated and replenished with fresh medium containing a corresponding concentration of AICAR or Compound C, and challenged with control vehicle or 100 ng/ml LPS for a further 24 h. At the end of the experiment, organ weights were recorded, and the accumulation of IL-1β (a, d, g), IL-6 (b, e, h) and IL-8 (c, f, i) was measured in supernatants by ELISA. Data are presented as mean concentration per mg tissue + SEM from 4 independent experiments, and analyzed by ANOVA using Dunnett’s multiple comparisons test to compare with vehicle (0), within each treatment group; * P < 0.05, ** P < 0.01, *** P < 0.001, NS = ANOVA not significant. (PDF) [file pone.0151416.s001.pdf]

S1 Fig

Manipulation of AMPK activity regulates inflammation in endometrial tissue

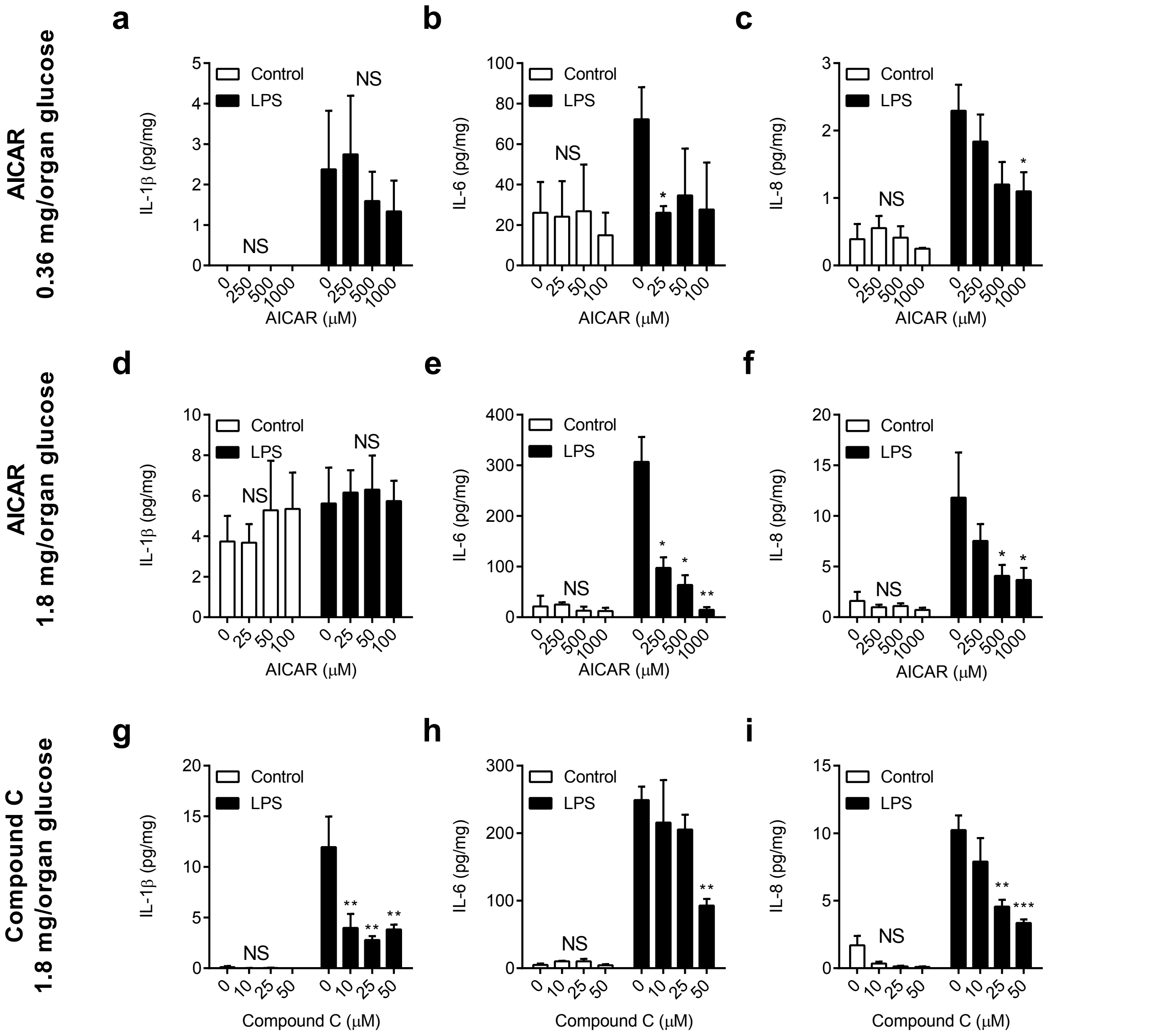

Ex vivo organ cultures of endometrium were cultured for 6 h in medium containing 0.36 mg/organ glucose with AICAR (a-c: 0, 250, 500, 1000 μM), 1.8 mg/organ glucose with AICAR (d-f: 0, 250, 500, 1000 μM), or 1.8 mg/organ glucose with compound C (g-i: 0, 10, 25, 50 μM). Media was then aspirated and replenished with fresh medium containing a corresponding concentration of AICAR or Compound C, and challenged with control vehicle or 100 ng/ml LPS for a further 24 h. At the end of the experiment, organ weights were recorded, and the accumulation of IL-1β (a, d, g), IL-6 (b, e, h) and IL-8 (c, f, i) was measured in supernatants by ELISA. Data are presented as mean concentration per mg tissue + SEM from 4 independent experiments, and analyzed by ANOVA using Dunnett's multiple comparisons test to compare with vehicle (0), within each treatment group; * $P < 0.05$, ** $P < 0.01$, *** $P < 0.001$, NS = ANOVA not significant.
