# Supplementary material for: Glucose Availability and AMP-Activated Protein Kinase Link Energy Metabolism and Innate Immunity in the Bovine Endometrium
Source: PLoS One. 2016 Mar 14;11(3):e0151416. doi: 10.1371/journal.pone.0151416 (PMC4790959; doi:10.1371/journal.pone.0151416)
Supplement: S2 Fig — Ex vivo organ cultures of endometrium were cultured in media containing 1.8 mg/organ glucose with (a-c) rapamycin or (d-f) torin-1 (0, 250, 500, 1000 nM) for 6 h followed by medium containing control vehicle or 100 ng/ml LPS for 24 h. At the end of the experiment organ weights were recorded, and the accumulation of IL-1β (a, d), IL-6 (b, e) and IL-8 (c, f) was measured in supernatants. Data are presented as mean concentration per mg tissue + SEM from 4 independent experiments. Data are presented as mean concentration per mg tissue + SEM from 4 independent experiments, and analyzed by ANOVA using Dunnett’s multiple comparisons test to compare with vehicle (0), within each treatment group; * P < 0.05, NS = ANOVA not significant. (PDF) [file pone.0151416.s002.pdf]

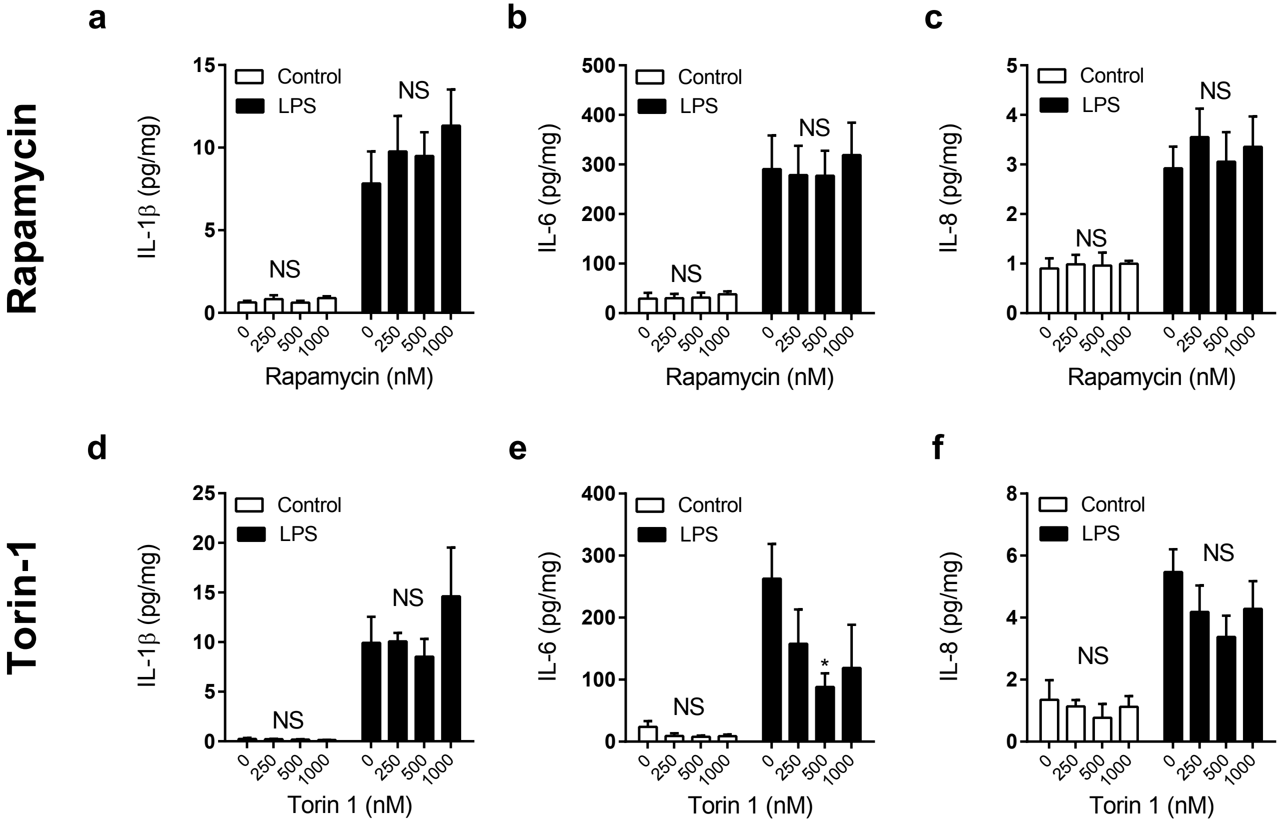

Ex vivo organ cultures of endometrium were cultured in media containing 1.8 mg/organ glucose with (a-c) rapamycin or (d-f) torin-1 (0, 250, 500, 1000 nM) for 6 h followed by medium containing control vehicle or 100 ng/ml LPS for 24 h. At the end of the experiment organ weights were recorded, and the accumulation of IL-1β (a, d), IL-6 (b, e) and IL-8 (c, f) was measured in supernatants. Data are presented as mean concentration per mg tissue + SEM from 4 independent experiments. Data are presented as mean concentration per mg tissue + SEM from 4 independent experiments, and analyzed by ANOVA using Dunnett's multiple comparisons test to compare with vehicle (0), within each treatment group; * $P < 0.05$, NS = ANOVA not significant.
