# Supplementary material for: Glucose Availability and AMP-Activated Protein Kinase Link Energy Metabolism and Innate Immunity in the Bovine Endometrium
Source: PLoS One. 2016 Mar 14;11(3):e0151416. doi: 10.1371/journal.pone.0151416 (PMC4790959; doi:10.1371/journal.pone.0151416)
Supplement: S3 Fig — Ex vivo organ cultures of endometrium were cultured in media containing 1.8 mg/organ glucose with IGF-1 (0, 25, 50, 100 ng/ml) for 6 h, followed by challenge with medium containing control vehicle or 100 ng/ml LPS for 24 h. At the end of the experiment organ weights were recorded and the accumulation of IL-1β (a), IL-6 (b) and IL-8 (c) was measured in supernatants. Data are presented as mean concentration per mg tissue + SEM from 4 independent experiments, and analyzed by ANOVA; NS = not significant. (PDF) [file pone.0151416.s003.pdf]

S3 Fig

IGF-1 does not modulate inflammation in endometrial tissue

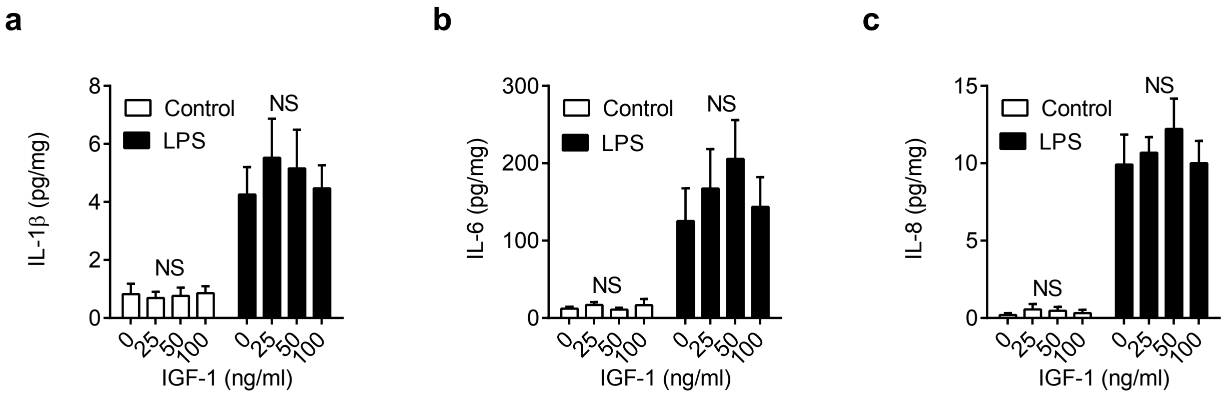

Ex vivo organ cultures of endometrium were cultured in media containing 1.8 mg/organ glucose with IGF-1 (0, 25, 50, 100 ng/ml) for 6 h, followed by challenge with medium containing control vehicle or 100 ng/ml LPS for 24 h. At the end of the experiment organ weights were recorded and the accumulation of IL-1β (a), IL-6 (b) and IL-8 (c) was measured in supernatants. Data are presented as mean concentration per mg tissue + SEM from 4 independent experiments, and analyzed by ANOVA; NS = not significant.
